# A two-genome microarray for the rice pathogens *Xanthomonas oryzae *pv. *oryzae *and *X. oryzae *pv. *oryzicola *and its use in the discovery of a difference in their regulation of *hrp *genes

**DOI:** 10.1186/1471-2180-8-99

**Published:** 2008-06-18

**Authors:** Young-Su Seo, Malinee Sriariyanun, Li Wang, Janice Pfeiff, Jirapa Phetsom, Ye Lin, Ki-Hong Jung, Hui Hsien Chou, Adam Bogdanove, Pamela Ronald

**Affiliations:** 1 Department of Plant Pathology, University of California, Davis, CA 95616, USA; 2 Department of Plant Pathology, Iowa State University, Ames, IA 50011, USA; 3 ArrayCore Facility, School of Veterinary Medicine, Molecular Biosciences, University of California, Davis, CA 95616, USA; 4 Department of Computer Science, Iowa State University, Ames, IA 50011, USA; 5 Department of Genetics, Development and Cell Biology, Iowa State University, Ames, IA 50011, USA; 6 Department of Biochemistry and Molecular Biology, Medical University of South Carolina, Charleston, SC 29425, USA

## Abstract

**Background:**

*Xanthomonas oryzae *pv. *oryzae *(*Xoo*) and *X. oryzae *pv. *oryzicola *(*Xoc*) are bacterial pathogens of the worldwide staple and grass model, rice. *Xoo *and *Xoc *are closely related but *Xoo *invades rice vascular tissue to cause bacterial leaf blight, a serious disease of rice in many parts of the world, and *Xoc *colonizes the mesophyll parenchyma to cause bacterial leaf streak, a disease of emerging importance. Both pathogens depend on *hrp *genes for type III secretion to infect their host. We constructed a 50–70 mer oligonucleotide microarray based on available genome data for *Xoo *and *Xoc *and compared gene expression in *Xoo *strains PXO99^A ^and *Xoc *strain BLS256 grown in the rich medium PSB vs. XOM2, a minimal medium previously reported to induce *hrp *genes in *Xoo *strain T7174.

**Results:**

Three biological replicates of the microarray experiment to compare global gene expression in representative strains of *Xoo *and *Xoc *grown in PSB vs. XOM2 were carried out. The non-specific error rate and the correlation coefficients across biological replicates and among duplicate spots revealed that the microarray data were robust. 247 genes of *Xoo *and 39 genes of *Xoc *were differentially expressed in the two media with a false discovery rate of 5% and with a minimum fold-change of 1.75. Semi-quantitative-RT-PCR assays confirmed differential expression of each of 16 genes each for *Xoo *and *Xoc *selected for validation. The differentially expressed genes represent 17 functional categories.

**Conclusion:**

We describe here the construction and validation of a two-genome microarray for the two pathovars of *X. oryzae*. Microarray analysis revealed that using representative strains, a greater number of *Xoo *genes than *Xoc *genes are differentially expressed in XOM2 relative to PSB, and that these include *hrp *genes and other genes important in interactions with rice. An exception was the *rax *genes, which are required for production of the host resistance elicitor AvrXa21, and which were expressed constitutively in both pathovars.

## Background

The rice pathogens *Xanthomonas oryzae *pathovar *oryzae *(*Xoo*) and *Xanthomonas oryzae *pathovar *oryzicola *(*Xoc*) cause economically significant disease in many rice-growing regions of the world [1]. *Xoo *invades rice vascular tissue to cause bacterial leaf blight, whereas *Xoc *colonizes the mesophyll parenchyma tissue to cause bacterial leaf streak. *Xoo *gains access to the xylem through wounds or natural openings such as hydathodes, while *Xoc*, in contrast, enters the leaf mainly through stomata [2]. *Xoo *and *Xoc *are closely related, infect the same host, and are often both established in the same rice fields. The complete genome sequences of Japanese *Xoo *strain T7174 (also called MAFF311018) and Korean *Xoo *strain KACC10331 have been published [3,4]. The genome sequences of a third *Xoo *strain, Philippine strain PXO99^A^, and a strain of *Xoc*, Philippine strain BLS256, have recently been completed and are also publicly available, through the Comprehensive Microbial Resource ([5]; GenBank Accession CP000967). The genomes of *Xoo *and *Xoc *strains are similar with respect to size, % G+C, and gene content, but show several inversions and rearrangements and some indels relative to one another (P. Patil and AJB, unpublished). These bacteria constitute an excellent comparative model for understanding determinants of tissue specificity in plant-bacterial interactions. Defining differences in gene expression and gene regulation between *Xoo *and *Xoc *is an important step toward that goal.

DNA microarray technology makes it possible to monitor the expression of thousands of genes simultaneously. Microarrays can be of two general types: 1) arrays based on *in situ *synthesis of oligonucleotide probes, using photochemical techniques or an ink-jet oligonucleotide synthesizer [6,7] and 2) spotted arrays, consisting of presynthesized DNA molecules or oligomers deposited onto glass slides or filter membranes [8,9]. Spotted arrays are generally less costly to produce, and because they are spotted rather than synthesized from a template, they constitute a highly flexible design platform.

Currently, oligonucleotide- or amplicon-spotted microarrays representing the whole or partial genomes of the following plant pathogenic bacteria are available: *Pseudomonas syringae*, *Ralstonia solanacearum*, *Xanthomonas axonopodis*, *Xanthomonas campestris*, and *Xylella fastidiosa *[10-16]. These arrays, enabled by whole-genome sequence availability, have been used to study responses to environmental cues such as heat shock [13], and to probe gene expression patterns related to pathogenesis [10,12,15]. They have also been used to assess genome diversity of isolates of a particular organism by comparative genome hybridization [11,17].

Based on the available genome sequences, we constructed a combined *Xoo *and *Xoc *whole genome microarray for both pathovars of *X. oryzae *(*Xo*) that contains 4,676 distinct 50–70 mer oligonucleotides, representing sequences from 2,153 genes shared by *Xoo *and *Xoc*, sequences specific to 1,270 *Xoo *genes, sequences specific to *Xoc *1,252 genes, and a control corresponding to a gene encoding hygromycin phosphotransferase not found in *Xoo or Xoc*. We present here the details of the microarray design and optimization, and the results of a successful experiment to validate the array by comparing gene expression of strain PXO99^A ^of *Xoo *and strain BLS256 of *Xoc *(hereafter "*Xoo*" or "*Xoc*" will be used to refer to these specific strains, unless otherwise indicated) in a rich medium vs. XOM2, a minimal medium reported to induce the *hrp *(hypersensitive reaction and pathogenesis) genes in *Xoo *strain T7174 [18,19], and by independently assessing the expression of a subset of those genes by semi-quantitative RT-PCR.

In addition to several arbitrarily selected genes, validation by semi-quantitative RT-PCR focused on the *hrp *and *rax *genes. The *hrp *genes encode a type III secretion system (T3SS), which many plant pathogenic bacteria, including *Xoo *and *Xoc*, require for *h*ypersensitive *r*eaction elicitation in resistant or non-host plants and for *p*athogenesis in susceptible host plants [20-22]. Expression of *hrp *genes is regulated by plant signals as well as in response to environmental stimuli such as carbon source, temperature, and pH [23,24]. The *rax *genes are *r*equired for AvrXa21 activity. AvrXa21 is a pathogen associated molecule recognized by the Xa21 resistance protein [25]. *rax *genes include eight genes predicted to contribute to three roles: type I secretion, sulfur metabolism and two-component regulation [25-28].

## Results and discussion

### Quality of the Xo array

**Figure 1:**
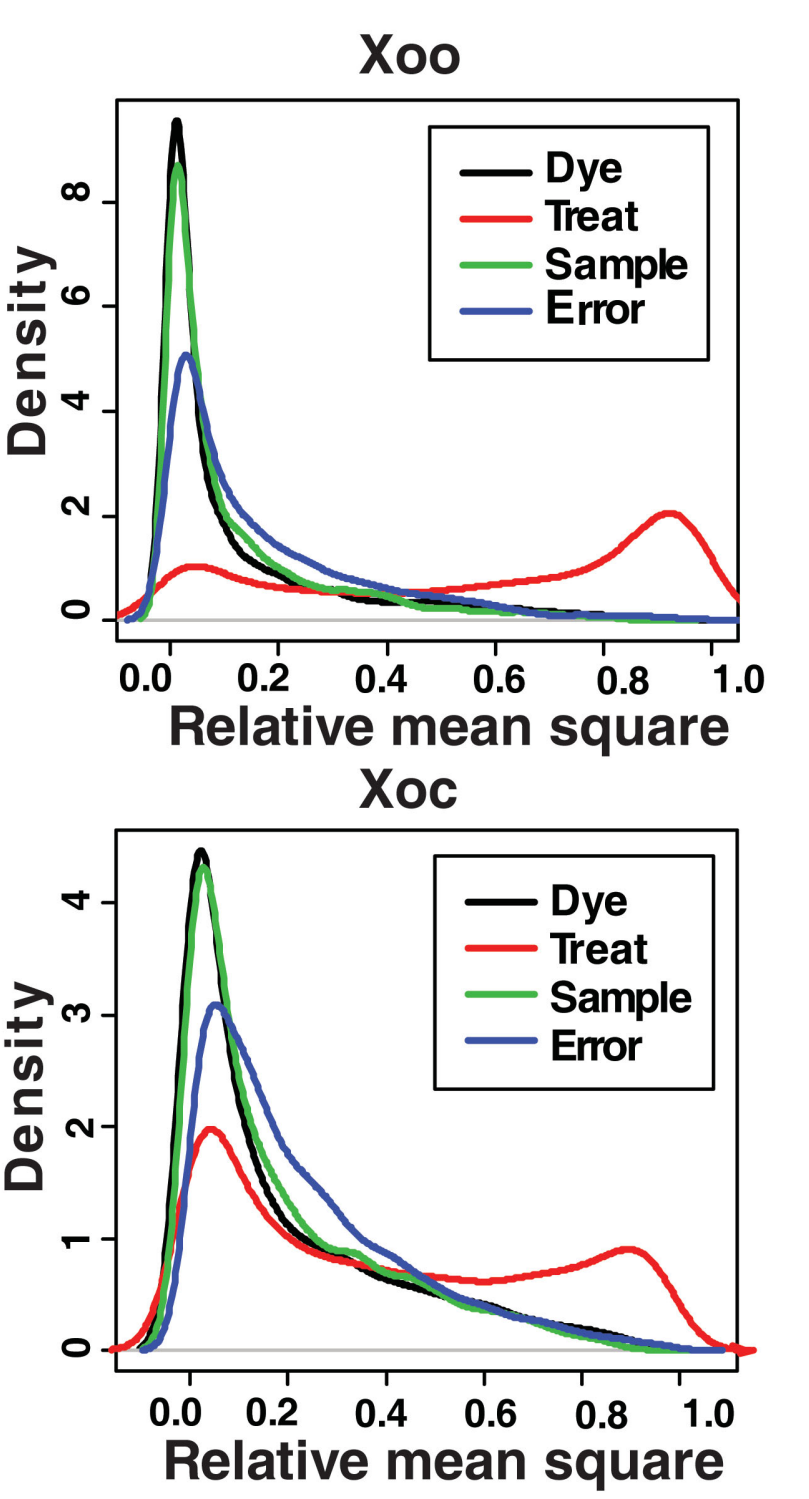
**Cumulative frequency distribution chart of sources of variation in the microarray data.** The four different factors, including treatment (XOM2 vs. PSB; treat), RNA sample (sample), Cy5 or Cy3 dye (dye), and unknown sources of variation (error), were considered for this ANOVA analysis. The significance of each factor across the array was evaluated as a frequency distribution of relative mean square values. The frequency was marked as a density on the Y axis. Relative mean square values of the four factors for all genes were obtained and correspond to the significance of the factor, *i.e*., a larger relative mean square value indicates that the factor is more significant.

We used a diagnostic method developed by Rocke [29] to examine the contribution of different factors to measured differences in signal intensity in 6 hybridizations (3 replicates with dye-swaps) comparing *Xoo *gene expression in PSB vs. XOM2 using the *Xo *array. This method employs analysis of variance (ANOVA). ANOVA can be used to calculate the gene expression changes in replicated array experiments and to correct systematic errors [30]. Factors examined were treatment, dye, sample, and error (Fig. 1). The treatment factor (red line) had the largest effect whereas the dye, sample, and error effects were much smaller. This analysis indicates that the measured significant changes in gene expression are due to the treatment and not to variability of other parameters.

The non-specific background error rate was assessed using 100 replicate spots of an oligonucleotide probe corresponding to the non-*Xo *gene encoding hygromycin phosphotransferase (*hph*). Across 6 hybridizations each using cDNA prepared from *Xoo *or *Xoc *RNA (3 replicates for each pathovar, each with a dye swap), error rates for positive artifacts were 0.00–1.00% and 1.00–2.00% respectively. That is, less than 2% of the *hph *gene oligonucleotides showed over two-fold differences in signal intensity. Error rates associated with non-spotted controls (632 empty spots) were similarly low, 0.00–0.94% and 0.00–1.10% following hybridization with *Xoo*- and *Xoc*-derived cDNA, respectively (Additional File 1).

### Optimization of hybridization temperature and sample amounts

To optimize temperature and amount of labeled cDNA sample for hybridization, array performance was assessed at 42, 44, 48, and 52°C and using labeled cDNA amounts of 10, 20, 30, 40, and 50 pmol. The mean signal intensity associated with the cyanine dyes and the correlation coefficients in self-self hybridizations were examined. There were no differences in these parameters associated with using high vs. low power of the scanner photomultiplicator (PMT). Hybridization with probe (labelled cDNA amounts of 50 pmol gave the best correlation coefficient values (0.93). For temperature, the best correlation coefficient (0.87) was obtained at 42°C. A hybridization temperature of 42°C and a labeled cDNA amount of 50 pmol resulted in the strongest signals associated with the cyanine dyes (data not shown) and the highest correlation coefficients among arrays (Table 1). Therefore, these parameters were used in all subsequent hybridizations.

**Table 1:** Optimization of hybridization temperature and probe amounts

Amount of probe^a ^(pmol)	10	20	30	40	50
HighPMT^b^	0.69^c^	0.70	0.70	0.92	0.93
LowPMT	0.72	0.73	0.71	0.93	0.94

Hybridization temperature (°C)	42	44	48	52	

HighPMT	0.87	0.73	0.67	0.77	
LowPMT	0.87	0.72	0.73	0.76	

^a ^Probes were generated from RNAs extracted from *Xoo *cultured in PSB.

^b ^PMT represents power of the scanner photomultiplicator that can influence ratio experimental estimation, dynamical range extension or saturation of highly expressed genes. In this study, we tuned to two (high and low) PMT levels to acquire raw slide pictures.

^c ^value of correlation coefficient in self-self hybridization.

### Application and validation of the array to identify *Xoo *and *Xoc *genes differentially expressed in a rich vs. a minimal medium

Given the distinct tissue specificities of *Xoo *and *Xoc*, we reasoned that these two pathovars might regulate the expression of important pathogenesis-associated genes differently. Therefore, we used the microarray to assess whether *Xoo *and *Xoc *show distinct patterns of differential gene expression in peptone sucrose broth (PSB) vs. XOM2, a minimal medium reported to activate *hrp *gene expression in *Xoo*, presumably by mimicking the pH and nutrient content in the apoplast [18]. Individually for *Xoo *and *Xoc*, three biological replicates (with a dye-swap, for a total of 6 hybridizations each) were carried out to compare gene expression in the two culture media. Average correlation coefficients across the biological replicates were 0.76 for *Xoo *and 0.69 for *Xoc*, respectively (Table 2.).

**Table 2:** Correlation coefficients of technical and biological replicates

Biological replicates	Pathovar	Test slide number						
		
		1 vs 2	1 vs 3	2 vs 3	4 vs 5	4 vs 6	5 vs 6	Average
Correlation coefficient	*Xoo*	0.73	0.72	0.63	0.90	0.66	0.92	0.76
	*Xoc*	0.71	0.71	0.72	0.66	0.67	0.66	0.69

Technical replicates	Pathovar	Test slide number						
		
		1 vs 4	2 vs 5	3 vs6				Average
				
Correlation coefficient	*Xoo*	0.85	0.91	0.91				0.89
	*Xoc*	0.65	0.77	0.67				0.70

^a ^Correlation coefficient of *Xoo *samples.

^b ^Correlation coefficient of *Xoc *samples.

To identify differentially expressed genes, the LMGene Package [29] was used. The resulting list of genes with significantly different expression between the two growth conditions was then refined using a false discovery rate (FDR) of 5% and a fold-change minimum of 1.75 (log2ratio value > 0.8), resulting in 247 genes for *Xoo *and 39 genes for *Xoc*. Additional File 2 provides a complete list of the differentially expressed *Xoo *and *Xoc *genes, sorted according to functional category and fold-change in expression (log2ratio).

**Figure 2:**
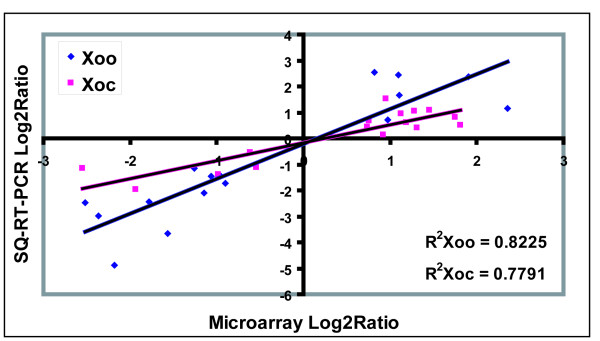
**Comparison of transcription measurements by microarray and semi-quantitative RT-PCR assays in *Xoo *and *Xoc*.** The relative transcriptional levels for the 16 genes of *Xoo *and *Xoc *were determined by microarray and semi-quantitative RT-PCR. The semi-quantitative RT-PCR log2ratio values were plotted against the microarray data log2ratio values. The correlation coefficients (R^2^) between the two dataset are 0.8225 and 0.7791 for *Xoo *and *Xoc *respectively.

To validate these results, semi-quantitative RT-PCR was used to independently assess expression levels for 16 *Xoo *and 16 *Xoc *genes selected arbitrarily from the list (genes and primer sets used are given in Table 3, and semi-quantitative RT-PCR results are shown in Additional file 3). RNA samples that were used in the microarray experiment as well as RNA samples extracted from three additional replicate sets of cultures were used as templates. There was good correlation between the semi-quantitative RT-PCR and the microarray results (correlation coefficients were 0.8225 and 0.7791 for *Xoo *and *Xoc *genes, respectively, Fig. 2). Although the amplitude of gene expression fold change between the two techniques is different, as might be expected since semi-quantitative RT-PCR is not a reliable measure of quantitative differences, the general trend of gene expression is consistent. For additional verification, we performed quantitative RT-PCR on 5 genes from *Xoo *and 2 from *Xoc*. In each case the results verified the expression patterns observed using semi-quantitative RT-PCR (Additional file 4).

**Table 3:** Sequences of forward (F) and reverse (R) primers used in semi-quantitative RT-PCR to validate *Xoo *and *Xoc *gene expression changes determined by microarray analysis.

GeneID^a^	Primer Sequence
XOO4289	F 5' ACA TCG CCG ATA ATT TCC AG 3' R 5' CGC AAC ACC TTG TAC TCG AC 3'
XOO4035	F 5' GGT CTT CGG ATC GTC AAC AT 3' R 5' GAT CAG AAA GCC GAT CTT GC 3'
XOO1994	F 5' GTT GGA GCA CAC CAT GAA AG 3' R 5' GGT ACA GCT CCA GAC CGA TG 3'
XOO2803	F 5' CTG TTC CAA GCA GAC CCT GT 3' R 5' CAC GAT GGG AAA CCT GAA AC 3'
XOO0424	F 5' CGG CTG AAG AAC TAC GCT TC 3' R 5' CTT GGT CAG CTC GTT GAT GA 3'
XOO0423	F 5' CGA AGA AGG CCT CTA CAT GG 3' R 5' CGA AGA AGG CCT CTA CAT GG 3'
XOO0076	F 5' GTG CCA CGT TGA AGT CAA GA 3' R 5' CTC ACT TAA TTC GCG CTT CC 3'
XOO1379	F 5' GCG ATA CCA GTC CAG GAT GT 3' R 5' CTT TTC CTC GTT GCA CTG GT 3'
XOO0094	F 5' CAC CTA CGG CTT TGT CTG GT 3' R 5' CAT TGC CAA ATG TGT TGG AG 3'
XOO0770	F 5' ATC GGC AGG TCG TAC TTG AT 3' R 5' GTC AGA CCC TGC TGT TCT CC 3'
XOO0282	F 5' CTG ATG AAT GAG CCT CAC GA 3' R 5' GAT TCC ATG TAG CCC AGC AT 3'
XOO2163	F 5' AAC GGT AGA ACT TGC CAT CG 3' R 5' AAC CTG GAC ATC CTG GAC AT 3'
XOO2757	F 5' AGC GCA GTC GCT TAC CTT C 3' R 5' GCA TAC GAC GAC GAC TAC GA 3'
XOO1664	F 5' CAC GCG TCT ACT GGG AAG AT 3' R 5' AAC ACG TCA TAC AGC GCA TC 3'
XOO4468	F 5' ACG ATT TCG ACC TGG ACC AC 3' R 5' ACA AGG ACG CCG AAA AGA T 3'
XOO1458	F 5' CCA GCG TTC CAT CAC TAC G 3' R 5' AGG GTA ATT AAC CGG CTT CG 3'
XOCORF1456	F 5' AAT GAC AAT GAG GGC ATC AA 3' R 5' ACT GAT TTG CGT TGT CGT TG 3'
XOCORF3144	F 5' GCA GAC GTT CGA CAC TTT CA 3' R 5' GCC TGT GTC TGC GAC TTG TA 3'
XOCORF3137	F 5' ACG ACC GTA TCC AAC CAG AC 3' R 5' AAC ATG CTG CGG ATT TCT TC 3'
XOCORF2869	F 5' AGT CGT TCG TAC CAG CCA TC 3' R 5' GCT CAC CTC CTG CTT GTA GC 3'
XOCORF0857	F 5' GCC AGC TTG AAA GTC AGC TC 3' R 5' CAT TTG CAG CAT TGG TGA AG 3'
XOCORF0690	F 5' TTC CTT TTC GCC TGG AGT T 3' R 5' TTC ATC GAC ACC GTC ATT G 3'
XOCORF4060	F 5' AAG TCA GTC CCG GTC AAG GT 3' R 5' ATT CCT CCA CCA TCT CGT TG 3'
XOCORF0488	F 5' GAC GTT CCG ACC AAT CTG TT 3' R 5' CTG CCC GAT CTT GAT CAT CT 3'
XOCORF2820	F 5' AAG ACC ACC CAC AAG CTG TT 3' R 5' GAC CCT TGA TGG TCA GAT CG 3'
XOCORF4434	F 5' AAT CTG GCC AAC GTC GAT AC 3' R 5' AGC TGG ATC ATT TTC CAC CA 3'
XOCORF0026	F 5' ATG GTG GAA AGC CTC AAC AC 3' R 5' GCC AGG ATA TTG GTC TGG AA 3'
XOCORF0775	F 5' AAA CTC TCG TGC TTG GTG CT 3' R 5' CAG CGT ATT CGT AGG TGA CG 3'
XOCORF4022	F 5' CAG CAT TCG CTG AAG GAA CT 3' R 5' AAA TAC GGC ACC TTG TGC TC 3'
XOCORF3816	F 5' TAT ACT GGT CGC TGC TGG TG 3' R 5' CGG TAA GTC ACC TCG TAG CC 3'
XOCORF1384	F 5' CCA AGA TCC GCA AGA AGA AG 3' R 5' GGA TCA GCT TTT CGA TCT GC 3'
XOCORF2448	F 5' GCT CAC TTA ATT CGC GCT TC 3' R 5' AAC GAG CTG CTT AGC GTT GT 3'

^a^The gene ID is according to the primary annotation obtained from the Comprehensive Microbial Resource. XOO designations represent IDs of genes from *Xanthomonas oryzae *pv. *oryzae *KACC 10331 and XOCORF designations represent IDs of genes from *Xanthomonas oryzae *pv. *oryzicola *BLS256.

### *Xoo *and *Xoc *genes differentially expressed in XOM2 relative to PSB

**Figure 3:**
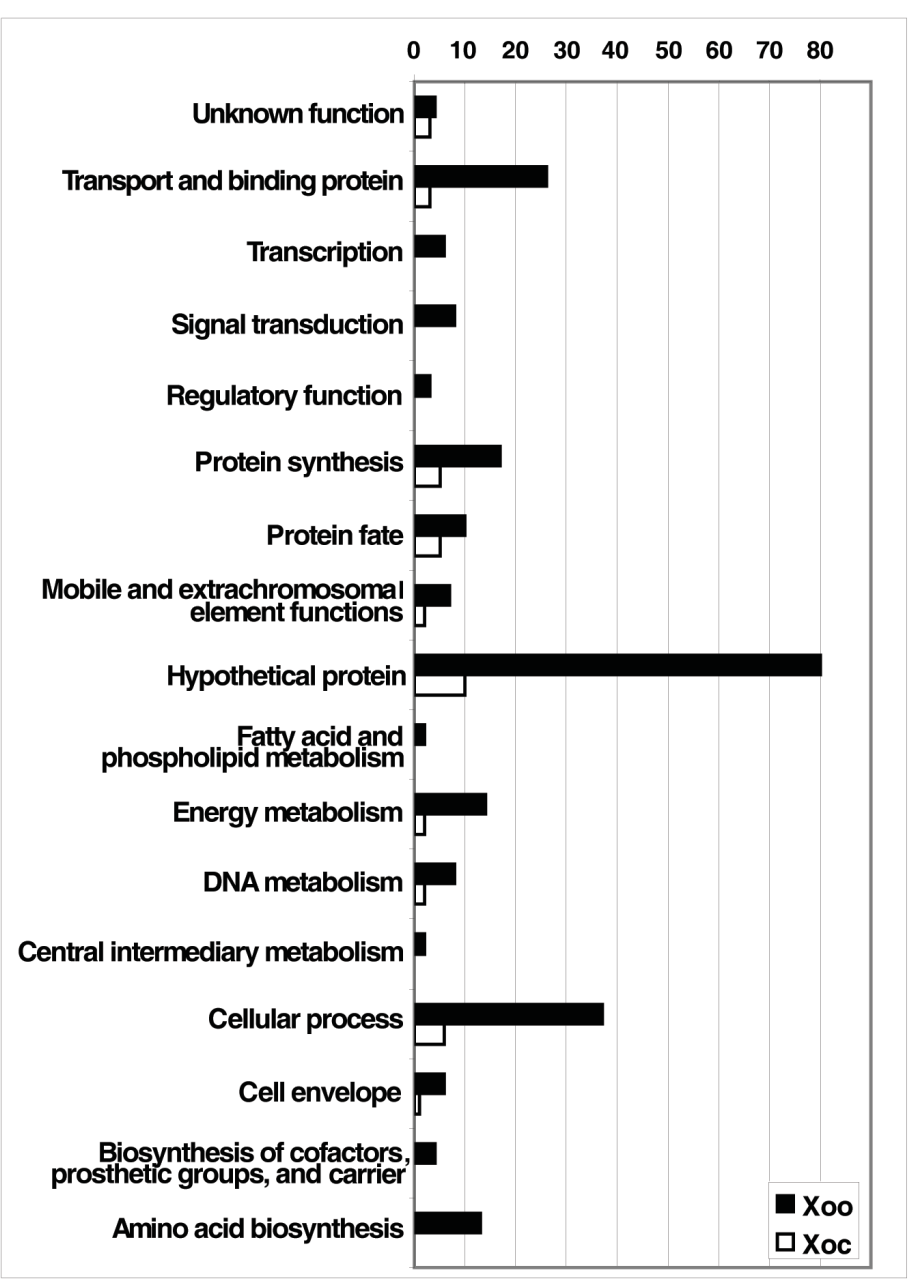
**Differentially expressed genes by functional category.** Functional categories are derived from the primary annotation retrieved from the TIGR Comprehensive Microbial Resource. Black bars indicate *Xoo *genes and white bars indicate *Xoc *genes.

Of the differentially expressed genes, 106 *Xoo *genes were up-regulated and 141 were down-regulated in XOM2 as opposed to PSB. For *Xoc*, only 28 and 11 genes were up-regulated and down-regulated, respectively, in XOM2 (Additional File 2). These genes represent 17 functional categories, based on the TIGR annotation for the genomes available through the Comprehensive Microbial Resource [5] (Fig. 3). The *Xoo *genes up-regulated in XOM2 encode primarily hypothetical proteins (29.2%) and proteins involved in cellular processes (22.6%); most of the down-regulated *Xoo *genes encode hypothetical proteins (34.8%) or proteins involved in signal transduction (5.0%), DNA metabolism (5.0%), mobile and extra-chromosomal element functions (4.3%), or transport and binding (12.1%). *Xoc *genes expressed at a higher level in XOM2 relative to PSB, as in *Xoo*, encode hypothetical proteins (28.6%) and proteins involved in cellular processes (21.4%). In contrast to *Xoo *however, *Xoc *genes down-regulated in XOM2 predominantly encode proteins involved in protein synthesis (45.5%).

#### Genes for general metabolism and transport and binding proteins

Many of the differentially expressed genes in *Xoo *and *Xoc *are involved in general metabolism, such as amino acid biosynthesis and energy metabolism. Also several genes for transport and binding proteins are differentially expressed in both strains. Differential expression of these genes likely reflects general adaptation to the different culture media related to nutrient uptake and utilization.

#### Chemotaxis and bacterial motility

A number of genes in *Xoo *and *Xoc *that are involved in motility and chemotaxis were up-regulated in XOM2 (Additional File 2), for example, in *Xoo*, chemotaxis genes *cheR *(XOO1466), encoding a methyl transferase, *cheW *(XOO1468), encoding a coupling protein, and *cheY *(XOO2622), encoding a two-component response regulator, and in *Xoc*, flagellar basal body and motor switch genes, *flgF *(XOCORF4434) and *fliN *(XOCORF4475). The expression of chemoreceptor genes *tsr *and *mcp *was up-regulated in response to XOM2 in both *Xoo *and *Xoc*. The chemoreceptors encoded by these genes perceive environmental chemicals and activate genes such as *pil *and *fli *that are involved in movement [31,32]. Consistent with this finding, *pil, fli*, and another gene involved in bacterial movement, *flg*, were also up-regulated in XOM2 both in *Xoo *and *Xoc*. These data are consistent with the fact that nutrient conditions, especially the type of carbon source, are involved in the regulation of bacterial motility [31-35]. Moreover, the abundance of genes involved in bacterial movement that are up-regulated in XOM2 suggests that *Xoo *and *Xoc *may activate genes for motility in the nutrient-limited environments of the rice xylem and mesophyll tissue and that these genes may be important for virulence. This notion is supported by the recent report that mutations in genes for twitching motility resulted in reduced virulence in *Xoc *[36] and by the fact that bacterial chemotaxis has essential roles in virulence in *Ralstonia solanacearum *[37].

#### Signal transduction genes

Two-component systems (TCS) are widespread signal transducers in prokaryotes that enable these organisms to respond to environmental stimuli through changes in gene expression [38]. Environmental cues are typically perceived through a sensor histidine kinase associated with the cell membrane. The second component is a response regulator, which upon activation by the sensor kinase activates downstream components of the response pathway. In many cases, signalling through a single two-component system results in a coordinated change in expression of multiple genes [39]. In *Xoo*, expression of the paired sensor kinase and response regulator genes *phoP *(XOO0423) and *phoQ *(XOO0424) is down-regulated in XOM2. *phoP*-*phoQ *is a two-component system that governs virulence, mediates the adaptation to Mg^2+^-limiting environment and regulates numerous cellular activities in *Salmonella *and other species [40,41]. We showed recently that *phoP *and *phoQ *in *Xoo *are required for activation of *hrp *genes and AvrXa21 activity, and full virulence [42]. Differential expression of *phoP-phoQ *was not detected in *Xoc*, raising the possibility that these genes are regulated differently by *Xoo *and *Xoc in planta *as well, and therefore may play a role in their distinctive pathogenicity. Another difference was a GGDEF domain protein (XOO2615) down-regulated in XOM2 in *Xoo *but not in *Xoc*. Recently, several GGDEF, EAL, and HD-GYP domain proteins of *X. campestris *were shown to play a role in virulence. They are hypothesized to compose a network of signal transduction systems for response to different environmental cues to modulate the level of the second messenger cyclic di-GMP [43].

#### rax genes (required for AvrXa21 activity)

We specifically examined expression levels of the *rax *(required for AvrXa21 activity) genes in *Xoo *and *Xoc*, and assessed expression of several of these genes independently by RT-PCR (Table 4) because AvrXa21 produced by *Xoo *is an important determinant for race-specific interactions and is postulated to be involved in bacterial cell-cell communication [25]. The *rax *genes are expressed constitutively in rich medium in *Xoo *[25]. They are highly conserved in *Xoo *and *Xoc*, but it is not known whether they are expressed in *Xoc *and whether *Xoc *produces AvrXa21. No significant differences in expression of any of the *rax *genes in *Xoo *and *Xoc *greater than 1.75 fold were observed in the microarray data, and the RT-PCR results confirmed this observation, except for *Xoo raxC *which showed a slight down regulation in XOM2. These results suggest that the expression of *rax *genes is largely unaffected by nutrient status. Their expression in *Xoc *raises the possibility that like *Xoo*, *Xoc *produces AvrXa21 or a similar molecule.

**Table 4:** Expression profiles of *hrp *and *rax *genes in *Xoo *and *Xoc *cultured in XOM2 vs. PSB by microarray and semi-quantitative RT-PCR analysis

Locus ID	Product	P-value^a^	Log2 ratio^a^	RT-PCR^b^	Locus ID	Product	P-value^a^	Log2 ratio^a^	RT-PCR^b^
XOO0095	*hpa1*	0	1.529		XOCORF2625	*hpa1*	0.000307	1.219	
XOO1379	*hrpG*	1.5E-09	1.102	+	XOCORF3400	*hrpG*	0.025035	0.086	0
XOO0094	*hrcC*	2.9E-11	1.094	+	XOCORF2442	*hpaF*	0.011623	0.019	
XOO0076	*hrpE*	1.1E-11	0.974	+	XOCORF2448	*hrpE*	0.010426	0.757	+
XOO1380	*hrpX*	0.000154	0.410	+	XOCORF3402	*hrpX*	0.0822	-0.443	0
XOO0085	*hrcU*	3.1E-06	0.313	+	XOCORF2466	*hpa2*	0.099664	-0.064	
XOO0090	*hrpB5*	0.345847	0.076		XOCORF2451	*hrcS*	0.000253	0.003	
XOO4533	*hrpB*	0.222464	0.063	+	XOCORF2289	*hrpB*	0.063541	0.073	0
XOO0087	*hrpB2*	0.411161	0.057		XOCORF2457	*hrpB2*	0.119757	0.013	0
XOO0075	*hpaB*	0.444516	0.041		XOCORF2447	*hpaB*	0.029266	0.194	
XOO0066	*hrpF*	0.792649	-0.019	+	XOCORF2444	*hrpF*	0.077523	0.036	0
XOO0079	*hpaA*	0.199054	-0.070	+	XOCORF2458	*hrpB3*	0.056872	0.026	
XOO0077	*hrpD6*	0.252583	-0.073	+	XOCORF2449	*hrpD6*	0.049163	-0.093	
XOO0089	*hrpB4*	0.01751	-0.152		XOCORF2459	*hrpB4*	0.45174	-0.038	
XOO0091	*hrcN*	4.1E-05	-0.196		XOCORF2462	*hrpB7*	0.039424	-0.237	
XOO0083	*hpaP*	0.004091	-0.202		XOCORF2454	*hpaP*	0.061767	-0.008	

XOO3396	*raxQ*	0.407272	-0.038		XOCORF3280	*raxQ*	0.045558	0.088	0
XOO3544	*raxA*	0.807527	-0.014	0	XOCORF1002	*raxA*	0.274492	0.057	
XOO3535	*raxR*	0.418945	0.056		XOCORF0993	*raxR*	0.786683	0.015	0
XOO3397	*raxP*	0.000764	0.183	0	XOCORF3279	*raxP*	0.014682	-0.018	0
XOO3534	*raxH*	0.001411	-0.191		XOCORF0994	*raxH*	0.319727	-0.054	
XOO0927	*raxC*	0.001717	-0.352	-	XOCORF3127	*raxC*	0.123998	-0.162	0
XOO3545	*raxST*	4.2E-05	-0.288	0					
XOO3543	*raxB*	0.422388	-0.051	0					

^a^Results of microarray analysis, as described in text. A log2 ratio of 0.8 is equivalent to a 1.75 fold relative increase in expression.

^a^Results of RT-PCR, as described in text. +, up-regulated in XOM2; -, down-regulated in XOM2; 0, no change in transcript abundance detected, blank, not tested.

#### hrp genes (hypersensitive reaction and pathogenicity)

We also examined expression of *hrp *genes, which as described above, are essential for pathogenicity in both *Xoo *and *Xoc*. Expression of *Xoo hpa1 *(XOO0095), *hrpG *(XOO1379), *hrcC *(XOO0094), and *hrpE *(XOO0076) were up-regulated in XOM2, consistent with the report by Tsuge et al. [18] which showed that several *hrp *loci in *Xoo *strain T7174 are induced in XOM2. No other *Xoo hrp *genes represented on the array showed induction greater than 1.75 fold in XOM2. However, when several were examined by semi-quantitative RT-PCR each was detectably up-regulated (Table 4). The Tsuge *et al*. study reported >200 fold induction for some *hrp *genes in XOM2. It should be noted, however, that the authors used a GUS reporter, and therefore measured accumulated enzyme activity, which does not necessarily correlate quantitatively with microarray results, which measure accumulation of mRNA. Also, there may be differences in the response of the two strains, T7174 and PXO99^A^, used here, to XOM2. Clearly though, semi-quantitative RT-PCR appears to have been a more sensitive assay for some of the *hrp *genes represented on the array. In stark contrast to the results with *Xoo*, in *Xoc*, expression increase of greater than 1.75 fold in XOM2 in the microarray experiment was only observed for *hpa1 *(XOCORF2625). Upregulation was not detected for any of several *Xoc hrp *genes tested by semi-quantitative RT-PCR with the exception of *hrpE*, for which increased expression in XOM2 was detectable by this method. Xiao *et al *[44], using RT-PCR, observed *hpa1 *expression in strain RS105 of *Xoc *in a synthetic medium they named XOM3, but not in nutrient broth (NB). XOM3 is identical to XOM2 except that it substitutes Fe(II)-EDTA for Fe(III)-EDTA. The authors also reported expression of the *gfp *gene cloned downstream of the *hrpX *promoter in cultures grown in XOM3 but not NB. The reporter construct itself was positioned downstream of the *lac *promoter, so this finding is difficult to interpret, but may indicate a difference in *hrp *regulation between RS105 and BLS256, the strain used here. Unfortunately, no other *hrp *genes were tested in RS105.

Our observation that, in *Xoc *cultured in XOM2, *hrp *genes other than *hpa1 *and *hrpE*, were not induced, including genes encoding the key Hrp regulators HrpG, a member of the OmpR family of response regulators of two-component systems [45], and HrpX, an AraC-type transcriptional activator that is the target of HrpG [46] underscores the fact that *hrp *genes are regulated differently in *Xoo *vs. *Xoc*, and reveals that the differences reside at or upstream of *hrpG*, perhaps at the level of environmental sensing. This *in vitro *finding in turn suggests that *hrp *gene expression may differ for these pathogens in *in planta *environments (e.g., the xylem and the mesophyll apolast), an exciting possibility that remains to be tested, and that may provide clues to tissue specificity.

The fact that *Xoc hpa1 *and *hrpE *are induced in XOM2, despite lack of induction of *hrpG*, *hrpX*, and other *hrp *genes, indicates that *hpa1 *and *hrpE *are under different or additional regulatory controls from the other *hrp *genes. Curiously, both genes encode extracellular proteins. HrpE is the main structural component of the *hrp *pilus [47], and Hpa1 is a Hrp-secreted protein with similarity to harpins [48], glycine-rich proteins that may assist in type III delivery of effectors into plant cells [49]. The differential regulation we have detected here for *hpa1 *and *hrpE *may reflect differential regulation *in planta*. This possibility fosters the intriguing speculation that the corresponding proteins accumulate early in the plant-bacterial interaction for rapid deployment once the rest of the type III secretion apparatus is assembled.

Tsuge et al (2006) [19] demonstrated that induction of *hrpG *in XOM2 was partially dependent on the *trh *(transcriptional regulator of *hrp*) gene, and that *trh *was also required for wildtype levels of *hpa1 *expression *in planta*, but that *trh *mutation did not result in a measurable difference in virulence. In light of our uncoupling of *hpa1 *and *hrp *expression in *Xoc*, it may be informative to assay the effect of the *trh *mutation in *Xoo *on the expression of other *hrp *genes *in planta*. Clearly, however, the possibility of multiple pathways for activation of *hrp *gene expression under different conditions exists.

## Conclusion

In contrast to other large scale approaches to the study of gene expression in plant pathogenic bacteria, including cDNA-AFLP [50] and *in vivo *expression technology (IVET, [51-53]), the whole genome microarray allows for genome-wide profiling of transcript levels under different conditions and over time. Cost, flexibility, sensitivity, and specificity are important factors that affect the utility of an array. In this study, we designed and constructed a microarray for *Xoo *and *Xoc *based on spotted 50–70-mer oligonucleotides. This platform is a relatively low cost and flexible, with good sensitivity [54]. Using the PICKY software, we were able to maximize specificity of probes on the array.

Our initial experiments with the *Xo *array and validation of select gene expression values by semi-quantitative RT-PCR demonstrate that the array generates robust and reliable data, though it may not be as sensitive as RT-PCR for some genes. By comparing gene expression in *Xoo *and *Xoc *cultured in PSB vs. XOM2, these experiments also provide insight and prompt new hypotheses regarding differential regulation of genes between *Xoo *and *Xoc *that may contribute to their distinct pathogenic characteristics. It is important to note that the artificial minimal medium XOM2 cannot be presumed to be an accurate proxy for *in planta *conditions. For example, although we have demonstrated that the component(s) or properties of XOM2 that induce *in vitro hrp *gene expression in *Xoo *are not effective for *Xoc*, it is not clear whether these component(s) or properties are the same as those that induce *Xoo hrp *gene expression *in planta*, or whether in fact *Xoo *and *Xoc *respond to different, or identical, cues in the host. Nonetheless, the results presented provide several candidate genes whose expression it will be important to compare *in planta*, and whose regulation it will be important to elucidate, toward gaining a detailed understanding of *Xoo *and *Xoc *pathogenicity that can then be used to develop more effective and environmentally-sound disease management practices.

## Methods

### Bacterial strains, growth conditions, and media

*Xanthomonas oryzae *pv. *oryzae *strain PXO99^A ^(Philippine race 6 provided by Jan Leach) and *X. oryzae *pv. *oryzicola *strain BLS256 were used for these experiments. Cells were grown at 28°C with shaking at 200 rpm, in nutrient-rich PSB (10 g/liter of peptone, 10 g/liter of sucrose, 1 g/liter of L-glutamic acid, monosodium salt; [28]). For experiments testing the effects of the modified minimal medium, XOM2, bacterial cells were cultured in PSB until OD600 equaled 0.2, washed twice, and then immediately transferred into XOM2 for 16 hrs. XOM2 [18] consists of 0.18% xylose sugar, 670 μM D, L-methionine, 10 mM sodium L(+)-glutamate, 14.7 mM KH_2_PO_4_, 40 μM MnSO_4_, 240 μM Fe(III). EDTA and 5 mM MgCl_2_, pH 6.5. Cells were washed twice prior to being harvested.

### RNA preparation

RNA was isolated using TRIzol^® ^reagent (Invitrogen, Carlsbad, CA, U.S.A.). The RNA samples were treated with 10 units of DNaseI (Invitrogen, Carlsbad, CA, U.S.A) for 30 min at room temperature, followed by column purification using the RNeasy midi kit (Qiagen, Germantown, MD, U.S.A.). The quality of RNA was determined by carrying out gel electrophoresis on a 1% agarose gel and was verified visually by using an Agilent 2100 Bioanalyzer (Agilent Technologies, Santa Clara, CA, U.S.A.). The quantity of total RNA was determined by measuring the absorbance at 260 nm and 280 nm. In addition, the level of protein contamination in the RNA was measured by using A260/A280 ratio.

### cDNA generation and labeling

cDNA was generated by using SuperScript™ III First-Strand kit and following the manufacturer's protocol (Invitrogen, Carlsbad, CA, U.S.A.). Twenty micrograms of high quality RNA was used mixed with random hexamers that were used as primers for cDNA generation and the mixture then preheated at 70°C for 15 mins. Primers were annealed to total RNA and extended with a labeling mixture consisting of 6.0 μl of 5× buffer, 2.0 μl of 0.1 M DTT, 1.0 μl of RNasin, 2.0 μl of SuperScript III reverse transcriptase, 2.0 μl of 25× dNTP-allyl-amino (aa) dUTP mixture (final concentration 0.5 mM each of dATP, cCTP, and dGTP, 0.35 mM aa-dUTP, 0.15 mM dTTP) at 25°C for 10 min followed by 2 h at 42°C. The RNA template was hydrolyzed using 3 μl of 2.5 N NaOH (37°C, 15 min) followed by neutralization with 15 μl of 2 M HEPES. Unincorporated primers and nucleotides were removed using the Zymo research kit according to the manufacturer's protocol (Zymo research, Genetix, UK) and the purified amino allyl-modified cDNA was resuspended in 60 μl of 50 mM sodium bicarbonate (pH 9.0). The amino allyl-modified cDNA was used to resuspend lyophilized Cy3 or Cy5 and incubated for 1 hr at room temperature in the dark. The reaction was quenched by adding 15 μl of 4 M hydroxylamine (15 min, room temperature in the dark). The dye-coupled cDNA was then purified by using the Zymo research kit (Zymo research, Genetix, UK).

### Oligo design

Our goal was to design a complete set of oligos that would uniformly detect gene-specific expression patterns for both *Xoo *and *Xoc*. To achieve the highest standard of uniformity, sensitivity and specificity for the *Xo *array it was necessary to utilize an optimized oligo design software that integrates the whole *Xo *gene set in its computation instead of considering each gene individually in a batch design mode. The *Xo *array carries 2 copies of the combined oligonucleotide set chosen by PICKY, the most efficient software developed to date, for this task [55].

The combined oligo set was designed based on the following steps, using PICKY for each oligo selection step: 1) The gene sets of *Xoo *and *Xoc *were combined with an additional hygromycin phosphotransferase gene and given to PICKY as a whole to design the shared oligos, i.e., oligos that can target at least one *Xoo *and one *Xoc *gene; 2) *Xoo *and *Xoc *genes targeted by the chosen shared oligos were removed from their individual gene sets and served as the second round design nontargets, i.e., genes that should be avoided by any PICKY designed oligo; 3) PICKY was then used to design oligos that can identify the remaining *Xoo *genes, using the earlier removed *Xoo *genes and the hygromycin phosphotransferase gene as nontargets; 4) Similarly, PICKY was used again to design oligos that can identify the remaining *Xoc *genes, using the removed *Xoc *genes and the hygromycin phosphotransferase gene as nontargets; 5) Finally, all oligos designed in steps 1, 3 and 4 were merged together to form the combined 4,675 oligo set.

Genome sequences and primary annotation of *Xoo *and *Xoc *were retrieved from the Comprehensive Microbial Resource [5] version 2.3 on December 22, 2005. The annotation is provided as Additional files 5 and 6. For *Xoo *KACC10331, these data are also available from the NCBI GenBank, under accession NC_006834. For *Xoc *BLS256, the finished genome sequence is also available from the NCBI Genbank, under accession AAQN01000001, but at the time of writing, the annotation has not yet been accessioned in that database.

### Oligo synthesis

Oligos used for spotting were synthesized by Integrated DNA Technology [56]. As a control, an oligo was designed by PICKY to detect *hph *but not *Xo *genes. This control was included in 2 well positions on each 384-well oligo synthesis plate except the last plate, which had only one *hph *position. Because the positions of this control were randomized across plates, the oligo also served as a check for array printing when labelled and hybridized against the array.

### Spotting

Microarrays were prepared at the ArrayCore Microarray Facility at the University of California, Davis [57]. Oligonucleotides were suspended in 1× Nexterion Spot solution at a final concentration of 20 μM and spotted onto aminosilane coated glass slides (Schott-Nexterion, USA). Oligonucleotides were spotted using a Lucidea Array Spotter (Amersham) in a humidity controlled spotting chamber (70%) at room temperature. Microarrays were deposited using 190 μm column and row pitches, and spot diameters averaged 80 μm under these conditions. After spotting, slides were allowed to sit at 70% humidity overnight at room temperature to maximize oligo binding. Microarrays were allowed to dry at ambient conditions and stored in the dark under argon at room temperature until use. Slides and spotting plates were tracked using the array spotter's built-in barcode reader and the information was used to generate the gene array layout file of the spotted 5 k *Xo *oligonucleotide microarray.

### Amino-blocking pre-treatment

Prior to the hybridization process, oligo-spotted slides were pre-treated with a blocking step that removes unbound DNA-molecules and buffer substances from the slides by extensive washing in order to avoid any interference with subsequent hybridization experiments. Spotted slides were incubated in amino blocking solution (5 g succinic anhydride in 315 ml n-methylpyrrolidone, 35 ml 0.2 M sodium borate pH 8.0) at room temperature for 15 mins. Slides were placed in 0.1% SDS solution 20 sec and washed with nanopure water 20 sec two times and then were transferred into the sodium borohydride block solution to undergo a sodium borohydride pre-treatment.

### Sodium borohydride pre-treatment

To minimize non-specific autofluorescence from the spotted material [58], slides were placed into a block solution containing 2× SSC, 0.05% SDS, 0.25% NaBH_4 _(Biochemical Technologies, USA) and incubated at 42°C for 20 min. Slides were transferred to 1× SSC for 5 min at room temperature and then sequentially washed with vigorous stirring using fresh 1× SSC (3 × 5 min, room temperature), 0.2× SSC (4 × 2 min, room temperature), and Nanopure water (1 × 2 min, room temperature). Slides were spin-dried (1000 rpm, 10 min) and stored under argon until use.

### Hybridization and scanning

Labeled probes were evaporated in a vacuum centrifuge on aqueous setting at 60°C to a volume of approximately 2–3 μl. Evaporated probes were then resuspended in 100 μl of a salt based hybridization solution (Ocimum Biosolutions) at room temperature. All hybridization and scanning steps were performed in a hepa and carbon filtered clean room at the ArrayCore Microarray Facility at University of California, Davis [57]. Hybridization occurred on a Tecan HS 4800 hybridization station. To block non-specific hybridization, a pre-hyridization buffer (5× SSPE, 6 M Urea, 0.5% Tween-20, 10× Denhardt's solution) was applied to the slides at 50°C and agitated for 15 minutes on the medium setting. Labeled probes were denatured by heating the mixture at 95°C for 3 mins and then snap-cooling on ice for 30 seconds. Probes were applied into the injector to hybridize with printed slides. Samples were hybridized for 16 hours at 42°C, Following hybridization, the slides were consecutively washed at 37°C with three salt based buffers of increasing stringency (2× SSC, 0.1% SDS, 1.0× SSC, and 0.5× SSC). Each buffer wash step was repeated twice, with a soak time of one minute followed by a one minute wash. A final wash step with water was performed. Following the final wash, slides were dried under a constant stream of N_2 _at 30°C. Slides were kept under N_2 _until scanning.

### Capture of raw data

Hybridized microarray slides were imaged using a GenePix 4000B dual laser microarray scanner (Axon Instruments, USA) at 5 μm resolution. Slides were imaged using 100% laser power for both lasers (532 nm and 635 nm) and scanned twice using the high PMT and low PMT settings. All images were processed using GenePix software (Axon Instruments, USA) for element identification and quantification. The metadata associated with the hybridizations, along with the "raw" intensities obtained from the GenePix quantitation.

### Validation of expression patterns of candidate genes using semi-quantitative RT-PCR and quantitative RT-PCR

For the first-strand cDNA synthesis, 100 ng of mRNA was reverse-transcribed in a total volume of 20 μl that contained 50 ng of random hexamer, 2.5 mM dNTP, 40 unit of RNaseOUT™, and 200 units of SuperScript™ III reverse transcriptase (the latter two components from Invitrogen, Carlsbad, CA) in reaction buffer supplied by the manufacturer. The reaction mixtures were incubated in 25°C for 10 min, then 4°C for 60 min. PCRs were performed in 50 μl reactions (containing 0.1 μl aliquots of the respective cDNA reaction mixture, 0.2 μM of gene-specific primers, 10 mM dNTPs, 1 unit of Taq DNA polymerase (Invitrogen), and 10× Taq buffer supplied by the manufacturer). Each reaction included an initial 5-min denaturation at 94°C, followed by 22 to 30 cycles of PCR (94°C, 45 sec; 60°C, 45 sec; 72°C, 45 sec), and a final 10 min at 72°C. Afterward, 20 μl of each reaction mixture was separated on a 1.0% agarose gel. (The primers used for semi-quantitative RT-PCR are described in Table 3). 16S ribosomal RNAs was used as controls for semi-quantitative RT-PCR. Primer sets used for semi-quantitative RT-PCR were designed using Primer3 [59]. Sequences of the primers used are shown in Table 3. Visualized band intensities of semi-quantitative RT-PCR products on the EtBr-stained agarose gels were transformed to digital values using Totallab TL100 software (Nonlinear Dynamics Ltd.). Log2 transformation was applied to digital band intensity values using the same mathematic transformation equations that had been applied to the microarray data. Fold changes from the microarray experiments were plotted against those from the semi-quantitative RT-PCR experiments.

For quantitative RT-PCR, cDNA generated as described in semi-quantitative RT-PCR was used as template for quantitative RT-PCR. Five *Xoo *and 2 *Xoc *genes were tested by using 50 μl reaction mixed with SYBR^® ^Green PCR Master Mix kit (Applied Biosystems, CA, USA.) and following the protocol as provided by manufacturer. Each reaction included an initial ramping 2 min 50°C, activation 10 mins 95°C, and then followed by 40 cycles of PCR (95°C, 15 sec; 60°C, 60 sec). The amount of *Xoo *and *Xoc *tested genes from PSB and XOM2 cultures were quantitated by calculating from each corresponding standard curves.

### Accession numbers

All of the microarray data have been deposited in the National Center for Biotechnology Information Gene Expression Omnibus (GEO) under accession number GSE 9658.

## Competing interests

The authors declare that they have no competing interests.

## Authors' contributions

Y–SS, MS, AB, PR designed the research project. Y–SS, YL, K–HJ, HHC constructed the microarray. MS, LW, Y–SS, JP^1 ^prepared samples for microarray studies. MS, Y–SS, JP^3^, JP^1^ performed the microarray experiments. Y–SS, MS analyzed microarray data. Y–SS, MS, LW, JP^5^, HC, AB, PR drafted the manuscript.

## Supplementary Material

Additional file 1 Error rate for *Xo *microarray hybridized with labeled cDNA derived from *Xoo *or *Xoc *RNA using a heterologous gene (*hph*) and empty spots. Click here for file

Additional file 2 *Xoo *and *Xoc *genes differentially expressed in XOM2 relative to PSB by microarray analysis using a false discovery rate of 5% and a fold-change minimum of 1.75 (log2ratio 0.8). Click here for file

Additional file 3 Validation of microarray results using semi-quantitative RT-PCR. 16s rDNA (16sDNA) was used as a control. The log2 ratios are shown for the expression of 16 select genes in *Xoo *(A) and *Xoc *(B) cultured in XOM2 (X) vs. PSB (P), calculated based on densitometry of products separated by agarose gel electrophoresis and visualized by ethidium bromide staining. Click here for file

Additional file 4 Validation of microarray results using quantitative RT-PCR. Relative transcript levels of five *Xoo *(A) and two *Xoc *(B) genes in PSB vs. XOM2 culture were quantified with reference to corresponding standard curves and plotted as ng PCR product. The primers used are noted below each plot. Click here for file

Additional file 5 *Xanthomonas oryzae *pv. *oryzae *KACC10331 primary annotation retrieved from the public Comprehensive Microbial Resource version 2.3 on December 22, 2005. Click here for file

Additional file 6 *Xanthomonas oryzae *pv. *oryzicola *BLS256 primary annotation retrieved from the public Comprehensive Microbial Resource version 2.3 on December 22, 2005. Click here for file
