# Supplementary material for: A two-genome microarray for the rice pathogens Xanthomonas oryzae pv. oryzae and X. oryzae pv. oryzicola and its use in the discovery of a difference in their regulation of hrp genes
Source: BMC Microbiol. 2008 Jun 18;8:99. doi: 10.1186/1471-2180-8-99 (PMC2474671; doi:10.1186/1471-2180-8-99)
Supplement: Additional file 1 — Error rate for Xo microarray hybridized with labeled cDNA derived from Xoo or Xoc RNA using a heterologous gene (hph) and empty spots. [file 1471-2180-8-99-S1.doc]

**Additional File 1**. Error rate for *Xo* microarray hybridized with labeled cDNA derived from *Xoo* or *Xoc* RNA using a heterologous gene (*hph*) and empty spots.

| Slide | Pathovar | 1 | 2 | 3 | 4 | 5 | 6 |
| --- | --- | --- | --- | --- | --- | --- | --- |
| Error rate of non-specific hybridization between sample probes and *hph* spots^a^ (%) | *Xoo* | 1.00 | 0.00 | 0.00 | 1.00 | 1.00 | 0.00 |
|  | *Xoc* | 1.00 | 1.00 | 2.00 | 0.00 | 2.00 | 2.00 |
| Error rate of non-specific hybridization between sample probes and empty spots^b^ (%) | *Xoo* | 0.94 | 0.16 | 0.00 | 0.32 | 0.32 | 0.00 |
|  | *Xoc* | 0.16 | 0.16 | 0.32 | 0.00 | 0.00 | 1.10 |

^a^ The *hph* gene is from *E. coli*., and encodes hygromycin B phosphotransferase. The

number of *hph* spots in the array is 100.

^b^ The number of empty spots in the array is 632.
