# Supplementary material for: A two-genome microarray for the rice pathogens Xanthomonas oryzae pv. oryzae and X. oryzae pv. oryzicola and its use in the discovery of a difference in their regulation of hrp genes
Source: BMC Microbiol. 2008 Jun 18;8:99. doi: 10.1186/1471-2180-8-99 (PMC2474671; doi:10.1186/1471-2180-8-99)
Supplement: Additional file 2 — Xoo and Xoc genes differentially expressed in XOM2 relative to PSB by microarray analysis using a false discovery rate of 5% and a fold-change minimum of 1.75 (log2ratio 0.8). [file 1471-2180-8-99-S2.doc]

**Additional File 2.** *Xoo* and *Xoc* genes differentially expressed in XOM2 relative to PSB using a false discovery rate of 5% and a fold-change minimum of 1.75 (log2ratio 0.8)

| ***Xoo***  **Locus ID** | **P-value** | | **log2ratio (XOM2/PSB)** | | **Gene annotation** |
| --- | --- | --- | --- | --- | --- |
| ^1^**Amino acid biosynthesis** | | | | | |
| XOO0946 | 2.161E-13 | | 2.81 | | ketol-acid reductoisomerase (ilvC) |
| XOO0945 | 0.000E+00 | | 1.52 | | acetolactate synthase isozyme II large subunit (ilvG) |
| XOO2243 | 2.199E-05 | | 1.45 | | homoserine kinase (thrB) |
| XOO0942 | 7.320E-10 | | 1.40 | | threonine dehydratase catabolic (tdcB) |
| XOO0944 | 7.363E-15 | | 1.28 | | acetolactate synthase isozyme II small subunit (ilvM) |
| XOO2676 | 3.393E-10 | | 1.24 | | ornithine carbamoyltransferase (argF) |
| XOO1990 | 1.981E-10 | | 1.17 | | asparagine synthase B (asnB) |
| XOO3137 | 9.647E-14 | | 1.15 | | dihydrodipicolinate synthetase (dapA) |
| XOO2675 | 1.355E-13 | | 1.10 | | argininosuccinate synthase (argG) |
| XOO4333 | 6.317E-09 | | 0.89 | | 5-methyltetrahydropteroyltriglutamate-homocysteine methyltransferase (metE) |
| XOO2672 | 7.859E-08 | | 0.87 | | acetylglutamate kinase (argB) |
| XOO1893 | 4.154E-13 | | -1.15 | | peptidyl-prolyl cis-trans isomerase (slyD) |
| XOO2890 | 9.254E-07 | | -1.57 | | dihydrodipicolinate reductase (dapB) |
|  |  | |  | |  |
| **Biosynthesis of cofactors, prosthetic groups, and carrier** | | | | | |
| XOO0402 | 2.012E-09 | | 0.92 | | delta-aminolevulinic acid dehydratase (hemB) |
| XOO2987 | 7.093E-11 | | -0.85 | | biotin synthesis protein (bioC) |
| XOO3853 | 1.804E-08 | | -0.88 | | GTP cyclohydrolase II 3,4-dihydroxy-2-butanone 4-phosphate synthase (ribA) |
| XOO3854 | 1.175E-10 | | -0.94 | | riboflavin synthase alpha chain (ribE) |
|  |  | |  | |  |
| **Cell envelope** | | | | | |
| XOO1968 | 6.419E-06 | | 1.06 | | outer membrane antigen (oma) |
| XOO2296 | 1.090E-03 | | 0.96 | | lipid A 4-kinase (lpxK) |
| XOO4114 | 3.561E-11 | | -0.85 | | membrane protein (yiaA) |
| XOO0780 | 5.421E-06 | | -1.11 | | putative glycosyltransferase |
| XOO0595 | 2.363E-11 | | -1.27 | | phosphinothricin acetyltransferase (yncA) |
| XOO0781 | 0.000E+00 | | -2.04 | | putative glycosyl transferase (wxoC) |
|  |  | |  | |  |
| **Cellular process** | | | | | |
| XOO0282 | 4.109E-11 | | 2.36 | | cellulase (egl) |
| XOO1586 | 9.460E-13 | | 2.32 | | pilin (pilE) |
| XOO1160 | 1.929E-14 | | 1.75 | | fimbrial assembly protein (pilP) |
| XOO2622 | 0.000E+00 | | 1.73 | | chemotaxis protein (cheY) |
| XOO1585 | 0.000E+00 | | 1.65 | | fimbrial assembly protein (pilC) |
| XOO0095 | 0.000E+00 | | 1.53 | | hpa1 |
| XOO0417 | 3.036E-10 | | 1.46 | | catalase precursor (catB) |
| XOO1159 | 2.945E-14 | | 1.44 | | fimbrial assembly membrane protein (pilO) |
| XOO2606 | 1.670E-13 | | 1.42 | | flagellar protein (fliK) |
| XOO1419 | 4.751E-14 | | 1.38 | | twitching motility protein (pilU) |
| XOO3199 | 2.137E-13 | | 1.34 | | pilX |
| XOO4575 | 4.751E-11 | | 1.23 | | glutathione peroxidase (btuE) |
| XOO2949 | 8.263E-05 | | 1.22 | | cell division protein (hflB) |
| XOO2602 | 1.670E-13 | | 1.20 | | flagellar protein (fliG) |
| XOO2572 | 5.332E-13 | | 1.15 | | flagellar biosynthesis hook protein (flgE) |
| XOO2744 | 4.358E-12 | | 1.10 | | virulence regulator (xrvA) |
| XOO1379 | 1.478E-09 | | 1.10 | | hrpG |
| XOO2577 | 1.973E-08 | | 1.09 | | flagellar protein (flgI) |
| XOO0094 | 2.888E-11 | | 1.09 | | hrcC |
| XOO1468 | 8.937E-09 | | 1.01 | | chemotaxis protein (cheW) |
| XOO0076 | 1.136E-11 | | 0.97 | | hrpE |
| XOO2835 | 7.158E-09 | | 0.89 | | chemotaxis response regulator (cheY) |
| XOO1458 | 3.882E-07 | | 0.82 | | pirin-related protein |
| XOO1466 | 2.098E-09 | | 0.82 | | response regulator for chemotaxis (cheR) |
| XOO2558 | 4.993E-11 | | -0.82 | | chemotaxis protein (mcp) |
| XOO3179 | 1.026E-13 | | -0.86 | | gumB |
| XOO2758 | 3.211E-12 | | -0.86 | | stringent starvation protein B (sspB) |
| XOO0309 | 2.412E-12 | | -0.89 | | toluene tolerance protein (yrbE) |
| XOO4537 | 7.363E-15 | | -0.97 | | oxidoreductase (ucpA) |
| XOO2279 | 4.263E-14 | | -1.01 | | avirulence protein |
| XOO2738 | 6.101E-09 | | -1.05 | | virulence protein (acvB) |
| XOO1847 | 3.803E-14 | | -1.07 | | cobalt-zinc-cadmium resistance protein (czcD) |
| XOO2848 | 1.128E-12 | | -1.14 | | chemotaxis protein (tsr) |
| XOO4035 | 5.229E-14 | | -2.18 | | 1,4-beta-cellobiosidase |
| XOO1978 | 0.000E+00 | | -2.27 | | pili assembly chaperone |
| XOO1077 | 0.000E+00 | | -2.44 | | cellulase S |
| XOO4467 | 0.000E+00 | | -2.55 | | virulence associated protein (vapI) |
|  |  | |  | |  |
| **Central intermediary metabolism** | | | | | |
| XOO3399 | 7.643E-04 | | 1.04 | | sulfite reductase flavoprotein (xcysJ) |
| XOO3505 | 0.000E+00 | | -2.13 | | similar to sulfatase modifying factor 1 precursor |
|  |  | |  | |  |
| **DNA metabolism** | | | | | |
| XOO1414 | 8.786E-06 | | 0.96 | | histone-like protein (hup) |
| XOO1875 | 2.432E-09 | | -0.81 | | DNA ligase (lig2) |
| XOO2830 | 9.556E-12 | | -0.82 | | MotA protein (motA) |
| XOO3951 | 5.433E-12 | | -0.82 | | methyltransferase homolog M.XphI (xphIM) |
| XOO3461 | 1.929E-14 | | -0.89 | | type I restriction-modification system, R subunit (hsdR-2) |
| XOO2587 | 1.475E-13 | | -1.12 | | RNA polymerase sigma-54 factor (rpoN) |
| XOO3799 | 6.217E-14 | | -1.20 | | methyltransferase |
| XOO0770 | 3.803E-14 | | -1.57 | | ATP-dependent RNA helicase (rhlE) |
|  |  | |  | |  |
| **Energy metabolism** | | | | | |
| XOO3001 | 1.929E-14 | | 2.42 | | cytochrome C2 (cycA) |
| XOO1801 | 6.613E-14 | | 1.05 | | acyl-CoA dehydrogenase |
| XOO3412 | 1.739E-09 | | 1.00 | | fructose-bisphosphate aldolase |
| XOO0561 | 1.443E-05 | | 0.98 | | C-type cytochrome biogenesis protein (dsbD) |
| XOO3740 | 1.982E-09 | | 0.81 | | visC |
| XOO1071 | 1.020E-11 | | -0.86 | | methanol dehydrogenase regulator (moxR) |
| XOO4422 | 4.344E-10 | | -0.91 | | xylosidase arabinosidase (xylB) |
| XOO3236 | 2.458E-10 | | -0.97 | | NADH-ubiquinone oxidoreductase NQO7 subunit (nuoA) |
| XOO0891 | 1.838E-13 | | -0.97 | | propionate catabolism regulatory protein (prpR) |
| XOO0119 | 6.070E-11 | | -1.02 | | methylamine utilization protein precursor (mauG) |
| XOO0726 | 2.785E-12 | | -1.31 | | ATP synthase A chain (atpB) |
| XOO1225 | 0.000E+00 | | -1.37 | | cytochrome C6 |
| XOO3547 | 8.957E-11 | | -1.57 | | glycine decarboxylase (gcvP) |
| XOO3603 | 1.735E-11 | | -1.44 | | ribose-phosphate pyrophosphokinase |
|  |  | |  | |  |
| **Fatty acid and phospholipid metabolism** | | | | | |
| XOO2221 | 8.446E-08 | | 0.83 | | poly (3-hydroxybutyric acid) synthase (phbC) |
| XOO2123 | 0.000E+00 | | -1.61 | | acyl carrier protein phosphodiesterase (acpD) |
|  |  | |  | |  |
| **Hypothetical protein** | | | | | |
| XOO1796 | 0.000E+00 | | 2.66 | | hypothetical protein |
| XOO3088 | 0.000E+00 | | 2.65 | | conserved hypothetical protein |
| XOO4334 | 1.761E-12 | | 2.30 | | conserved hypothetical protein |
| XOO0704 | 1.791E-04 | | 1.63 | | conserved hypothetical protein |
| XOO1878 | 0.000E+00 | | 1.59 | | conserved hypothetical protein |
| XOO3411 | 1.382E-14 | | 1.45 | | conserved hypothetical protein |
| XOO2507 | 4.056E-12 | | 1.44 | | conserved hypothetical protein |
| XOO0735 | 1.200E-05 | | 1.39 | | conserved hypothetical protein |
| XOO1533 | 2.344E-10 | | 1.34 | | conserved hypothetical protein |
| XOO3200 | 2.724E-13 | | 1.31 | | conserved hypothetical protein |
| XOO3685 | 1.699E-13 | | 1.30 | | conserved hypothetical protein |
| XOO3479 | 9.021E-12 | | 1.27 | | conserved hypothetical protein (hcp) |
| XOO2984 | 6.132E-11 | | 1.17 | | conserved hypothetical protein |
| XOO0337 | 2.978E-06 | | 1.16 | | conserved hypothetical protein |
| XOO1803 | 1.569E-10 | | 1.05 | | conserved hypothetical protein |
| XOO1452 | 0.000E+00 | | 1.02 | | conserved hypothetical protein |
| XOO4195 | 4.077E-09 | | 0.95 | | conserved hypothetical protein |
| XOO2729 | 3.752E-10 | | 0.92 | | conserved hypothetical protein |
| XOO1805 | 2.578E-10 | | 0.91 | | conserved hypothetical protein |
| XOO3837 | 3.581E-05 | | 0.91 | | conserved hypothetical protein |
| XOO3481 | 1.748E-10 | | 0.90 | | conserved hypothetical protein |
| XOO0768 | 1.110E-08 | | 0.88 | | conserved hypothetical protein |
| XOO1131 | 4.001E-08 | | 0.88 | | conserved hypothetical protein |
| XOO0543 | 1.765E-07 | | 0.86 | | conserved hypothetical protein |
| XOO4617 | 9.654E-13 | | 0.85 | | conserved hypothetical protein |
| XOO2906 | 3.907E-08 | | 0.84 | | conserved hypothetical protein |
| XOO0523 | 4.312E-09 | | 0.84 | | conserved hypothetical protein |
| XOO4282 | 7.816E-08 | | 0.84 | | conserved hypothetical protein |
| XOO4241 | 5.911E-11 | | 0.82 | | conserved hypothetical protein |
| XOO1455 | 1.008E-07 | | 0.82 | | hypothetical protein |
| XOO1854 | 3.059E-11 | | 0.81 | | conserved hypothetical protein |
| XOO2059 | 9.354E-12 | | -0.82 | | conserved hypothetical protein |
| XOO3313 | 3.219E-10 | | -0.86 | | conserved hypothetical protein |
| XOO0422 | 1.232E-12 | | -0.87 | | conserved hypothetical protein |
| XOO0872 | 4.494E-12 | | -0.88 | | conserved hypothetical protein |
| XOO0421 | 6.282E-10 | | -0.88 | | conserved hypothetical protein |
| XOO2968 | 1.093E-05 | | -0.89 | | conserved hypothetical protein |
| XOO1070 | 3.719E-12 | | -0.90 | | conserved hypothetical protein |
| XOO4212 | 1.274E-13 | | -0.90 | | conserved hypothetical protein |
| XOO0725 | 1.939E-03 | | -0.90 | | conserved hypothetical protein |
| XOO4292 | 1.307E-12 | | -0.91 | | conserved hypothetical protein |
| XOO0513 | 2.185E-13 | | -0.91 | | conserved hypothetical protein |
| XOO3637 | 2.064E-13 | | -0.93 | | conserved hypothetical protein |
| XOO2354 | 1.506E-13 | | -0.93 | | conserved hypothetical protein |
| XOO3345 | 1.616E-09 | | -0.94 | | hypothetical protein |
| XOO3469 | 8.456E-13 | | -0.98 | | hypothetical protein |
| XOO0877 | 9.415E-08 | | -0.98 | | conserved hypothetical protein |
| XOO0870 | 1.157E-12 | | -0.99 | | conserved hypothetical protein |
| XOO4006 | 6.123E-07 | | -0.99 | | conserved hypothetical protein |
| XOO1806 | 2.783E-06 | | -1.02 | | conserved hypothetical protein |
| XOO1551 | 1.348E-12 | | -1.03 | | conserved hypothetical protein |
| XOO1420 | 0.000E+00 | | -1.04 | | conserved hypothetical protein |
| XOO3848 | 9.243E-09 | | -1.05 | | conserved hypothetical protein |
| XOO0036 | 5.450E-06 | | -1.05 | | conserved hypothetical protein |
| XOO4466 | 8.890E-12 | | -1.05 | | conserved hypothetical protein |
| XOO3221 | 5.009E-12 | | -1.07 | | conserved hypothetical protein |
| XOO2115 | 1.475E-08 | | -1.08 | | conserved hypothetical protein |
| XOO2402 | 1.004E-12 | | -1.08 | | conserved hypothetical protein |
| XOO2163 | 0.000E+00 | | -1.14 | | conserved hypothetical protein |
| XOO2585 | 1.852E-12 | | -1.15 | | conserved hypothetical protein |
| XOO3537 | 4.303E-12 | | -1.15 | | conserved hypothetical protein |
| XOO2714 | 1.214E-11 | | -1.18 | | conserved hypothetical protein |
| XOO3612 | 2.643E-12 | | -1.20 | | conserved hypothetical protein |
| XOO1687 | 1.503E-12 | | -1.24 | | hypothetical protein |
| XOO0593 | 4.834E-13 | | -1.24 | | conserved hypothetical protein |
| XOO2757 | 1.852E-12 | | -1.26 | | conserved hypothetical protein |
| XOO0871 | 3.357E-14 | | -1.26 | | conserved hypothetical protein |
| XOO0779 | 2.487E-14 | | -1.28 | | hypothetical protein (smtA) |
| XOO2864 | 1.238E-13 | | -1.30 | | conserved hypothetical protein |
| XOO3247 | 3.803E-09 | | -1.33 | | conserved hypothetical protein |
| XOO2162 | 7.363E-15 | | -1.37 | | conserved hypothetical protein |
| XOO0966 | 3.647E-12 | | -1.40 | | conserved hypothetical protein |
| XOO3508 | 0.000E+00 | | -1.45 | | conserved hypothetical protein |
| XOO2863 | 0.000E+00 | | -1.63 | | conserved hypothetical protein |
| XOO3126 | 6.613E-14 | | -1.64 | | conserved hypothetical protein |
| XOO2539 | 0.000E+00 | | -1.70 | | conserved hypothetical protein |
| XOO1664 | 1.503E-12 | | -1.78 | | conserved hypothetical protein |
| XOO4269 | 0.000E+00 | | -2.29 | | conserved hypothetical protein |
| XOO0869 | 0.000E+00 | | -2.35 | | conserved hypothetical protein |
| XOO1406 | 0.000E+00 | | -2.69 | | conserved hypothetical protein |
|  |  | |  | |  |
| **Mobile and extrachromosomal element functions** | | | | | |
| XOO1309 | 4.291E-08 | | 0.99 | | DNA transport competence protein (comEA) |
| XOO2381 | 2.761E-09 | | -0.81 | | putative ISXo8 transposase |
| XOO4183 | 2.591E-13 | | -0.99 | | putative IS1113 transposase (tnpX1) |
| XOO3084 | 8.018E-12 | | -1.02 | | putative ISXo8 transposase |
| XOO2470 | 6.933E-14 | | -1.33 | | putative transposase |
| XOO0674 | 0.000E+00 | | -1.38 | | putative transposase |
| XOO3076 | 0.000E+00 | | -2.23 | | possible transposase |
|  |  | |  | |  |
| **Protein fate** | | | | | |
| XOO1266 | 3.357E-14 | | 2.44 | | endoproteinase Arg-C |
| XOO3989 | 1.144E-05 | | 1.16 | | aminopeptidase (pepN) |
| XOO3281 | 2.379E-11 | | 1.10 | | metallopeptidase |
| XOO1510 | 2.172E-10 | | 1.01 | | major cold shock protein (cspA) |
| XOO3806 | 1.160E-08 | | 0.86 | | protease (xcp) |
| XOO1381 | 9.874E-14 | | -0.88 | | Hsp90xo protein (hsp90xc) |
| XOO4289 | 3.202E-11 | | -0.90 | | 10kDa chaperonin (groES) |
| XOO0059 | 6.247E-12 | | -1.04 | | protease DO (htrA) |
| XOO2375 | 0.000E+00 | | -1.76 | | serine protease |
| XOO1075 | 0.000E+00 | | -1.77 | | peptide deformylase (def) |
|  |  | |  | |  |
| **Protein synthesis** | | | | | |
| XOO4693 | 5.543E-08 | | 1.02 | | 23S ribosomal RNA |
| XOO4680 | 2.012E-09 | | 1.01 | | Glu tRNA |
| XOO4695 | 8.218E-08 | | 1.00 | | 16S ribosomal RNA |
| XOO4697 | 3.114E-08 | | 0.98 | | 5S ribosomal RNA |
| XOO2552 | 2.926E-13 | | 0.93 | | initiation factor IF-1 (infA) |
| XOO3568 | 1.351E-05 | | 0.84 | | 50S ribosomal protein L6 (rplF) |
| XOO4673 | 2.735E-06 | | 0.83 | | Asp tRNA |
| XOO3561 | 1.221E-10 | | 0.82 | | 30S ribosomal protein S13 (rpsM) |
| XOO4656 | 1.033E-06 | | 0.82 | | Ser tRNA |
| XOO0348 | 3.372E-13 | | -0.81 | | pseudouridylate synthase |
| XOO1153 | 4.281E-13 | | -0.89 | | 50S ribosomal protein L31 (rpmE) |
| XOO4649 | 1.967E-05 | | -0.99 | | Phe tRNA |
| XOO1327 | 1.827E-11 | | -1.08 | | tRNA (guanine-N1-)-methyltransferase (trmD) |
| XOO4667 | 1.209E-12 | | -1.14 | | Arg tRNA |
| XOO4666 | 7.759E-12 | | -1.25 | | Pro tRNA |
| XOO1328 | 0.000E+00 | | -1.33 | | 50S ribosomal protein L19 (rplS) |
| XOO4640 | 0.000E+00 | | -1.58 | | Gly tRNA |
|  |  | |  | |  |
| **Regulatory function** | | | | | |
| XOO1467 | 9.693E-13 | | 1.11 | | response regulator (vieA) |
| XOO0952 | 1.303E-07 | | 0.90 | | inorganic pyrophosphatase (ppa) |
| XOO0418 | 1.698E-11 | | 0.85 | | ankyrin-like protein (ankB) |
|  |  | |  | |  |
| **Signal transduction** | | | | | |
| XOO3405 | 2.186E-08 | | 0.97 | | sensor histidine kinase |
| XOO2615 | 1.728E-08 | | -0.88 | | GGDEF family protein |
| XOO2564 | 2.973E-11 | | -0.82 | | histidine kinase |
| XOO4201 | 3.874E-10 | | -0.92 | | two-component system sensor protein |
| XOO0424 | 2.631E-13 | | -0.97 | | two-component system sensor protein (phoQ) |
| XOO2241 | 3.601E-07 | | -1.01 | | tryptophan-rich sensory protein (tspO) |
| XOO0423 | 1.382E-14 | | -1.07 | | two-component system regulatory protein (phoP) |
| XOO2586 | 0.000E+00 | | -1.28 | | response regulator |
|  |  | |  | |  |
| **Transcription** | | | | | |
| XOO3142 | 0.000E+00 | | 0.95 | | transcriptional regulator |
| XOO3727 | 6.933E-14 | | -0.91 | | transcriptional regulator marR family |
| XOO0026 | 2.945E-14 | | -0.94 | | transcriptional regulator |
| XOO1998 | 6.111E-12 | | -0.95 | | transcriptional regulator marR family (yybA) |
| XOO0929 | 2.945E-14 | | -1.34 | | transcriptional regulator |
| XOO2803 | 7.363E-15 | | -2.37 | | transcriptional regulator tetR/acrR family |
|  |  | |  | |  |
| **Transport and binding protein** | | | | | |
| XOO3320 | 0.000E+00 | | 3.09 | | Oar protein (oar) |
| XOO1994 | 7.363E-15 | | 1.91 | | bacterioferritin (brf) |
| XOO2788 | 2.864E-05 | | 1.37 | | metabolite transport protein (yhjE) |
| XOO1596 | 2.487E-14 | | 1.29 | | competence lipoprotein (comL) |
| XOO4149 | 5.766E-12 | | 1.02 | | bacterioferritin (bfr) |
| XOO1778 | 3.092E-05 | | 1.01 | | colicin V secretion ABC transporter ATP-binding protein |
| XOO1669 | 1.544E-10 | | 0.86 | | outer membrane protein P6 precursor (ompP6) |
| XOO3749 | 7.459E-12 | | 0.84 | | apaG |
| XOO4582 | 4.017E-10 | | 0.82 | | outer membrane protein (ompP1) |
| XOO1958 | 1.178E-10 | | -0.84 | | probable ABC transporter ATP-binding protein |
| XOO0978 | 2.904E-11 | | -0.91 | | colicin I receptor (cirA) |
| XOO2205 | 1.332E-13 | | -0.92 | | manganese transport protein (smf2) |
| XOO0407 | 4.057E-13 | | -0.95 | | iron-uptake factor (piuB) |
| XOO2663 | 1.666E-13 | | -0.95 | | MFS transporter (cynX) |
| XOO0157 | 1.382E-14 | | -0.99 | | ABC transporter ATP-binding protein (tptC) |
| XOO1901 | 1.022E-07 | | -1.02 | | MFS transporter (araJ) |
| XOO2791 | 1.332E-13 | | -1.03 | | integral membrane transporter (yhjX) |
| XOO0470 | 6.164E-11 | | -1.09 | | Mg++ transporter (mgtE) |
| XOO4065 | 0.000E+00 | | -1.11 | | phaC |
| XOO2114 | 2.943E-13 | | -1.11 | | TonB-like protein |
| XOO2791 | 3.934E-12 | | -1.30 | | integral membrane transporter (yhjX) |
| XOO2805 | 0.000E+00 | | -1.36 | | multidrug efflux transporter (acrB) |
| XOO2804 | 1.648E-11 | | -1.45 | | multidrug resistance protein (acrA) |
| XOO1959 | 1.382E-14 | | -1.47 | | outer membrane receptor proteins, mostly Fe transport |
| XOO1405 | 0.000E+00 | | -2.02 | | ABC transporter permease (ynhE) |
| XOO1362 | 0.000E+00 | | -2.05 | | cation:proton antiporter (ybaL) |
|  |  | |  | |  |
| **Unknown function** | | | | | |
| XOO3424 | 1.198E-10 | | 0.95 | | unknown protein |
| XOO3458 | 3.357E-14 | | -0.88 | | Uncharacterized conserved protein |
| XOO0986 | 4.263E-14 | | -1.60 | | unknown protein |
| XOO4468 | 0.000E+00 | | -2.52 | | unknown protein |
|  |  | |  | |  |
| ***Xoc***  **Locus ID** | | **P-value** | | **log2 (XOM2/PSB)** | **Gene annotation** |
| **Cell envelope** | | | | | |
| XOCORF0775 | | 9.463E-04 | | -0.98 | rickettsia 17 kDa surface antigen family Rickettsia 17 kDa surface antigen family |
|  | |  | |  |  |
| **Cellular processes** | | | | | |
| XOCORF1519 | | 7.703E-04 | | 1.43 | osmotically inducible protein Y precursor (PA4739) |
| XOCORF0026 | | 2.556E-05 | | 1.18 | chemotaxis protein (tsr) |
| XOCORF4434 | | 1.884E-04 | | 0.95 | flagellar basal-body rod protein (flgF) |
| XOCORF4475 | | 8.000E-09 | | 0.90 | flagellar motor switch protein (fliN) |
| XOCORF0252 | | 1.607E-03 | | 0.89 | chemotaxis protein (mcp) |
| XOCORF2465 | | 3.068E-04 | | 1.22 | harpin (hpa1) |
|  | |  | |  |  |
| **DNA metabolism** | | | | | |
| XOCORF4487 | | 1.038E-04 | | 0.96 | RNA polymerase sigma factor (FliA) |
| XOCORF1698 | | 2.361E-03 | | -0.91 | ribonuclease BN (rbn) |
|  | |  | |  |  |
| **Energy metabolism** | | | | | |
| XOCORF3137 | | 1.542E-05 | | 1.28 | 2-isopropylmalate synthase (leuA) |
| XOCORF3589 | | 2.116E-03 | | 0.84 | acyl-CoA dehydrogenase (fadE) |
|  | |  | |  |  |
| **Hypothetical protein** | | | | | |
| XOCORF3584 | | 9.450E-02 | | 1.82 | hypothetical protein |
| XOCORF2604 | | 1.095E-05 | | 1.60 | conserved hypothetical protein |
| XOCORF2042 | | 6.440E-04 | | 1.27 | hypothetical UPF0337 protein |
| XOCORF2906 | | 1.771E-04 | | 1.23 | conserved hypothetical protein |
| XOCORF2599 | | 4.454E-04 | | 1.19 | conserved hypothetical protein |
| XOCORF2869 | | 4.672E-03 | | 1.13 | conserved hypothetical protein |
| XOCORF3687 | | 3.813E-05 | | 0.96 | hypothetical protein |
| XOCORF0035 | | 7.708E-04 | | 0.83 | hypothetical protein |
| XOCORF4021 | | 2.456E-04 | | -1.50 | hypothetical protein |
| XOCORF1456 | | 1.817E-01 | | -1.93 | conserved hypothetical protein |
|  | |  | |  |  |
| **Mobile and extrachromosomal element functions** | | | | | |
| XOCORF3808 | | 9.392E-03 | | 1.27 | ISRSO5-transposase protein (tISRso5) |
| XOCORF0286 | | 1.971E-05 | | 1.04 | IS1595 transposase |
|  | |  | |  |  |
| **Protein fate** | | | | | |
| XOCORF4060 | | 1.806E-04 | | 1.82 | DnaK supressor (dksA) |
| XOCORF0690 | | 1.305E-02 | | 1.75 | cysteine protease |
| XOCORF3810 | | 1.788E-01 | | 1.35 | major cold shock protein (cspA) |
| XOCORF0857 | | 1.548E-04 | | 1.31 | endoproteinase Arg-C |
| XOCORF1638 | | 2.361E-03 | | -1.33 | chaperonin GroEL |
|  | |  | |  |  |
| **Protein synthesis** | | | | | |
| XOCORF1230 | | 2.823E-02 | | -0.82 | ribosomal protein L24 (rplX) |
| XOCORF1236 | | 1.734E-03 | | -0.89 | ribosomal protein L22 (rplV) |
| XOCORF1232 | | 5.007E-04 | | -0.94 | 30S ribosomal protein S17 (rpsQ) |
| XOCORF1231 | | 3.813E-05 | | -1.04 | ribosomal protein L14 (rplN) |
| XOCORF1221 | | 7.703E-04 | | -1.09 | ribosomal protein L15 (rplO) |
|  | |  | |  |  |
| **Transport and binding proteins** | | | | | |
| XOCORF2576 | | 8.690E-03 | | 1.90 | entericidin A-related protein |
| XOCORF0488 | | 6.444E-04 | | 1.45 | sulfate transporter (PA0103)sulfate transporter |
| XOCORF3144 | | 9.463E-04 | | -2.55 | TonB dependent receptor domain protein |
|  | |  | |  |  |
| **Unknown function** | | | | | |
| XOCORF3678 | | 1.012E-04 | | 1.27 | protein of unknown function (DUF1328) superfamily |
| XOCORF2919 | | 1.806E-04 | | 1.11 | protein of unknown function (DUF520) superfamily |
| XOCORF2820 | | 1.735E-03 | | 0.92 | YceI like family family |

^1^ Genes are grouped according to functional category based on their annotation.
