# Supplementary figures and images for: A two-genome microarray for the rice pathogens Xanthomonas oryzae pv. oryzae and X. oryzae pv. oryzicola and its use in the discovery of a difference in their regulation of hrp genes
Source: BMC Microbiol. 2008 Jun 18;8:99. doi: 10.1186/1471-2180-8-99 (PMC2474671; doi:10.1186/1471-2180-8-99)

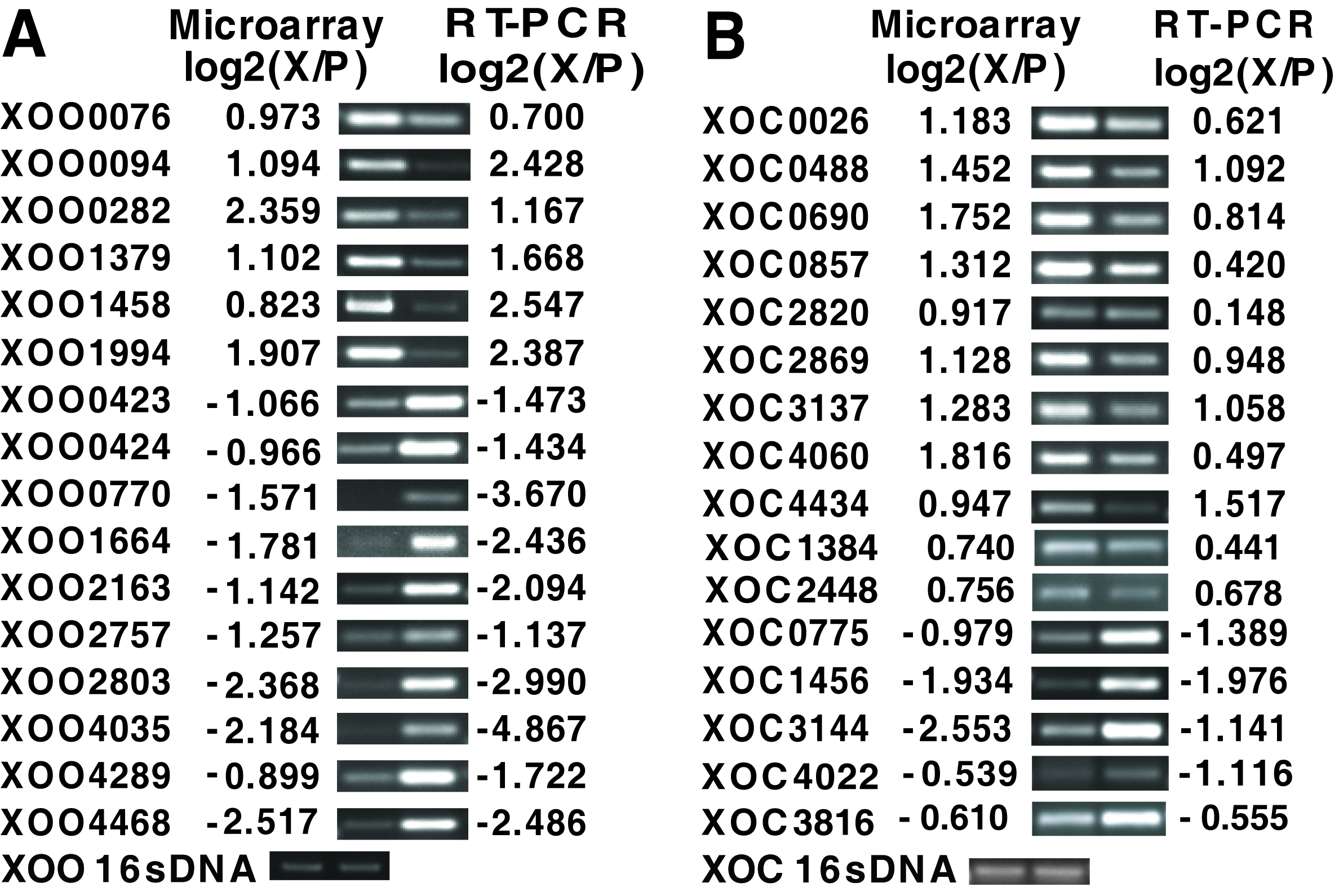

Supplement: Additional file 3 — Validation of microarray results using semi-quantitative RT-PCR. 16s rDNA (16sDNA) was used as a control. The log2 ratios are shown for the expression of 16 select genes in Xoo (A) and Xoc (B) cultured in XOM2 (X) vs. PSB (P), calculated based on densitometry of products separated by agarose gel electrophoresis and visualized by ethidium bromide staining. [file 1471-2180-8-99-S3.tiff]

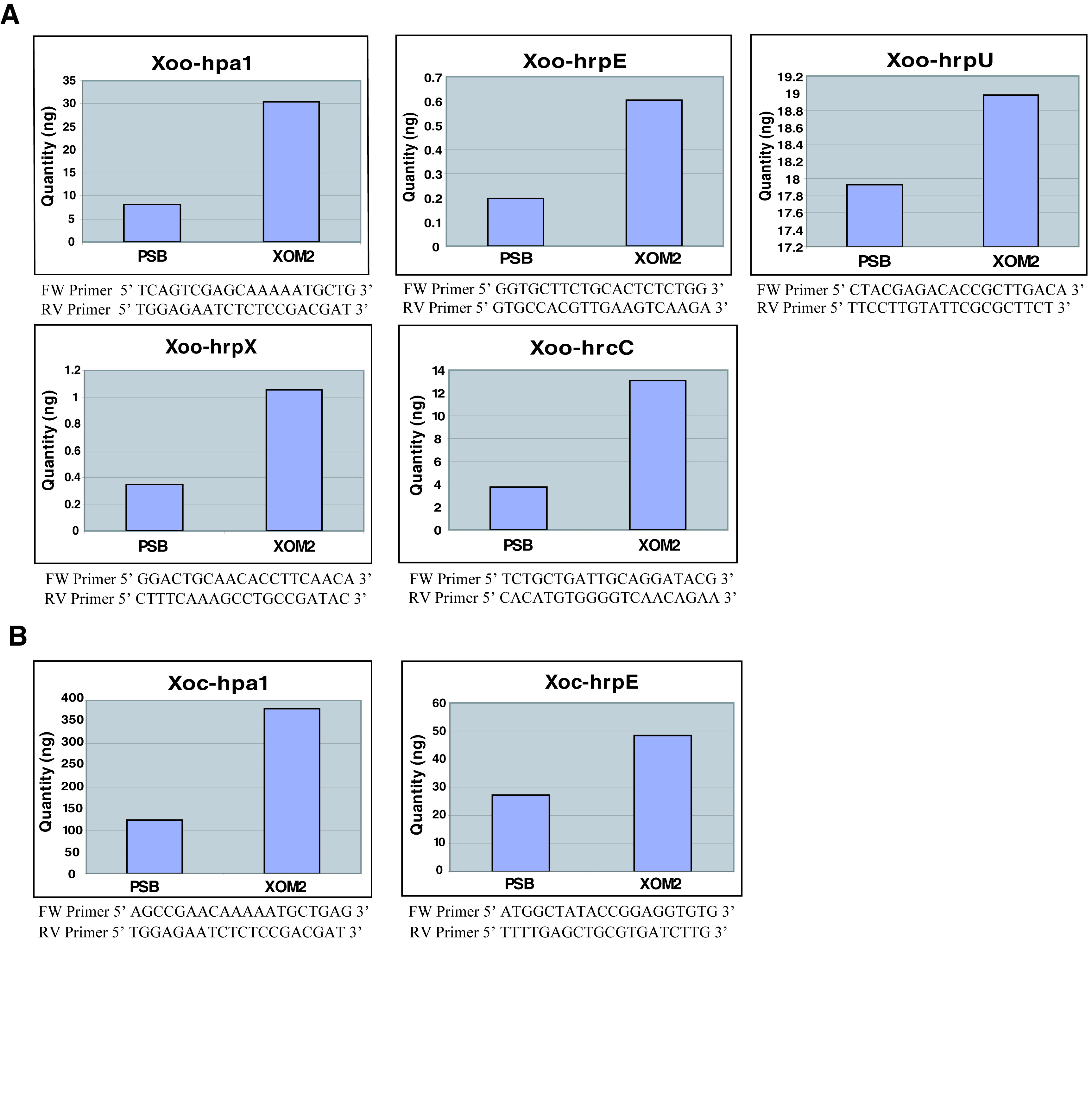

Supplement: Additional file 4 — Validation of microarray results using quantitative RT-PCR. Relative transcript levels of five Xoo (A) and two Xoc (B) genes in PSB vs. XOM2 culture were quantified with reference to corresponding standard curves and plotted as ng PCR product. The primers used are noted below each plot. [file 1471-2180-8-99-S4.png]
